# Volatilization and Retention of Metallic and Non-Metallic Elements During Thermal Treatment of Fly Ash

**DOI:** 10.3390/ma18061319

**Published:** 2025-03-17

**Authors:** Yegui Wang, Weifang Chen, Yifan Chen, Shuyue Zhang, Baoqing Deng

**Affiliations:** School of Environment and Architecture, University of Shanghai for Science and Technology, 516 Jun Gong Road, Shanghai 200093, China; 221580132@st.usst.edu.cn (Y.W.); chenweifang@usst.edu.cn (W.C.); 233401991@st.usst.edu.cn (Y.C.); 222291996@st.usst.edu.cn (S.Z.)

**Keywords:** fly ash, thermal treatment, heavy metal, volatilization, stabilization

## Abstract

This research investigated the volatilization and enrichment of metallic and non-metallic elements in municipal solid waste incineration fly ash during thermal treatment. The high-temperature treatment resulted in both the volatilization and stabilization of heavy metals in fly ash. The split of volatilization and stabilization depended highly on the original speciation. The results showed that loosely bound heavy metals were the main contributors to the leaching toxicity of the raw fly ash. These metals were also easily volatilized. The volatilization of heavy metals was accompanied by de-chlorination, indicating that the loss of heavy metals may be related to the evaporation of chloride compounds. On the other hand, heavy metals that were strongly bound with the fly ash were less volatile. For the six heavy metals investigated, 42% and 58% of Cd and Pb were volatilized at 800 °C. By comparison, the volatilizations of Cu, Zn, Cr, and Ni amounted to 18–31% at the same temperature. The remaining heavy metals became more stable. Stabilization could be attributed to reactions between decomposition products; thus, new and more complicated structures, such as Ca_3_Mg(SiO_4_)_2_, Ca_2_Al_2_SiO_7,_ and CuSiO_3,_ were formed. Heavy metals were incorporated into the structures and stabilized. Moreover, analyses of other elements showed that thermal treatment resulted in the enrichment of elements, including Mn, Mg, Si, and Al. This is conducive to reusing fly ash.

## 1. Introduction

As one of the byproducts of municipal solid waste incineration, fly ash is considered hazardous due to its contents of heavy metal and toxic organics. The amount of fly ash produced witnessed rapid growth with the increase in the amount of municipal solid waste (MSW) generated and in the share of incineration in MSW disposal [1]. The reuse and recycling of valuable materials from fly ash has become increasingly important for fly ash disposal and resource recovery [2]. Fan et al. [3] suggested that, since fly ash is rich in aluminosilicate, it could be reused as cementitious materials, soil amendments, or light-weight aggregates in construction and road pavement materials.

Thermal treatment is one of the most extensively investigated and widely employed technologies to detoxify fly ash [4,5]. It is renowned for its ability to stabilize heavy metals and degrade hazardous organics. It could also greatly reduce the volume of fly ash, thus easing the burden on disposal. Thermal treatment is classified into sintering (700–1200 °C), melting (1200–1600 °C) and vitrification (1100–1500 °C) based on the treatment temperature [6]. These treatments resulted in fly ash products with different particle sizes, crystalline structures, and morphologies [7].

Studies have shown that thermal treatment is a process that impacts the migration, stabilization, and reactions of heavy metals [8]. Heavy metal detoxification is partly caused by their migration from fly ash to the gaseous phase. Almost all Cu, Pb, Cd and up to 50% of Zn evaporated at temperatures between 1000 and 1100 °C [9]. Studies have shown that the presence of chlorides in fly ash facilitates volatilization [10,11]. Chlorides could react with oxides, such as SiO_2_ and Al_2_O_3_, to form chlorine gas, which, in turn, promote further volatilization [12]. Additionally, some of the heavy metals may exist as chloride salts which are readily volatile [13]. Therefore, the volatilization of heavy metal is often accompanied by de-chlorination.

On the other hand, thermal treatment is also an effective heavy metal solidification/stabilization method. Wang et al. [14] investigated the mineral transition behavior of fly ash at temperatures of between 300 and 1200 °C and reported that the crystal phases changed with the increase in temperature. Heavy metals were incorporated within the SiO_2_-Al_2_O_3_-CaO framework, which thus stabilized. Guo et al. [15] also proved that elements with high boiling points, Si, Al, Ca, etc., reacted under high temperatures to form stable silicoaluminates. These substances reduced the volatility of heavy metals such as Pb, Cr, Cu and Zn. There are also studies showing that the heavy metal elements in fly ash solidify through self-cementation via physical encapsulation [16,17]. Through the deconvolution of XPS spectra, Wan et al. [18] illustrated that the immobilization of Pb was attributed to the formation of PbO·3SiO_2_ and PbO·7SiO_2_.

Overall, complex chemical reactions occur during thermal treatment of fly ash, and both volatilization and stabilization have played a role in detoxification of heavy metals. Therefore, it is of interest to distinguish the behavior of metals during thermal treatment. Extreme volatilization of heavy metals could be problematic, as it causes secondary pollution. Understanding the behavior of heavy metals could help to improve thermal treatment through elements such as, for instance, adding additives to reduce volatilization. Furthermore, research on the thermal treatment of fly ash has heavily focused on heavy metals. Fly ash from the incineration of municipal solid waste is renowned for its complex chemical compositions. There are fewer studies on the thermal behavior of other metallic and non-metallic elements. The migration and transformation of these elements are also important both from the perspective of fly ash detoxification and resource recycling. It is necessary to investigate the thermal behaviors of fly ash comprehensively.

Therefore, the main purpose of this research is, first, to clarify the extent of volatilization and stabilization of heavy metals and other elements including Ca, Cl, Si, Mg, Al, etc. during thermal treatment at different temperatures. The connection between heavy metal volatilization and speciation was also explored. XRD patterns of fly ash before and after treatment were used to elucidate possible chemical reactions between heavy metals and mineral matrices. The ultimate goal was to provide detailed information on the behavior of fly ash during high-temperature treatment.

## 2. Materials and Methods

### 2.1. Fly Ash

The fly ash used in this study was from a municipal solid waste incineration plant in the Zhejiang Province of China. Raw fly ash (FA) was dried at 105 °C until it reached a constant weight before use.

### 2.2. Thermal Treatment

Thermal treatment was carried out in a tube furnace (OTF-1200X, Kejing, Hefei, China). The temperature was set from 500 °C to 1100 °C. Ten grams of fly ash were placed in a quartz boat (200 mm × 30 mm × 15 mm) and loaded into the furnace. The tube furnace was purged with N_2_ for 10 min before heating at a heating rate of 10 °C/min. Samples were heated to the predetermined temperature in N_2_ and kept at that temperature for 2 h before being cooled to room temperature. The gas flow rate was constant at 80 mL/min. The treated fly ash was named as a FA + temperature, such as FA800.

### 2.3. Characterization of Fly Ash

The thermal properties of fly ash under the N_2_ atmosphere were investigated via a TG/DSC Simultaneous Thermal Analyzer (TG/DSC, STA8000, PerkinElmer, Waltham, MA, USA) with a heating rate of 10 °C/min. X-ray Diffraction (XRD, Rigaku UItima IV, Tokyo, Japan) was employed to identify the crystal structures with a Cu-Kα radiation at 40 KV and 30 mA. 2θ was set at 5° to 90°. The chemical composition of fly ash was determined by an X-ray Fluorescence Spectroscopy (XRF-1800, Shimadzu, Tokyo, Japan).

### 2.4. Heavy Metal Analysis

The heavy metal contents of fly ash were measured based on a standard method of China (HJ 803-2016) [19]. Fly ash was digested with an acid mixture of aqua regia (HNO_3_:HCl = 1:3) for 2 h. The resultant mixture was then filtered through a 0.45 μm filter, and the filtrate was analyzed for heavy metals.

The speciation of heavy metals was obtained via a Tessier sequential extraction method [20]. Specifically, 2 g of fly ash was sequentially extracted via extractants of different strengths. Details on the protocol of extraction are outlined in Table S1 of the Supplementary Materials. After each extraction, the mixture was filtered through a 0.45 μm membrane filter and the filtrate was analyzed for heavy metals. The residual was then subjected to the subsequent extraction until the entire sequence was completed. The heavy metal fraction (in percentage) for each speciation was calculated by Equation (1).

(1) $$F\left(\%\right)=\frac{C_{m}V}{M_{t}}\;\times \;100$$

where F is the percentage of heavy metal speciation; C_m_ is the concentration of heavy metal in filtrate (mg/L); V is the volume of filtrate (L); and Mt is the total mass of heavy metals in the five speciations (mg).

The Acetic Acid Buffer Solution Method (HJ/T 300-2007) [21] was employed to investigate the heavy metal leaching toxicity. The initial pH of the acetic acid extraction solution was 2.88 ± 0.05. After 18 h of extraction, heavy metals in filtrate were analyzed for leaching toxicity.

The concentrations of heavy metals were determined by an Inductively Coupled Plasma Optical Emission Spectrometry (ICP-OES, Optima8000, PerkinElmer, Waltham, MA, USA).

## 3. Results and Discussion

### 3.1. Thermal Behavior via Thermogravimetric Analysis

**Figure 1:**
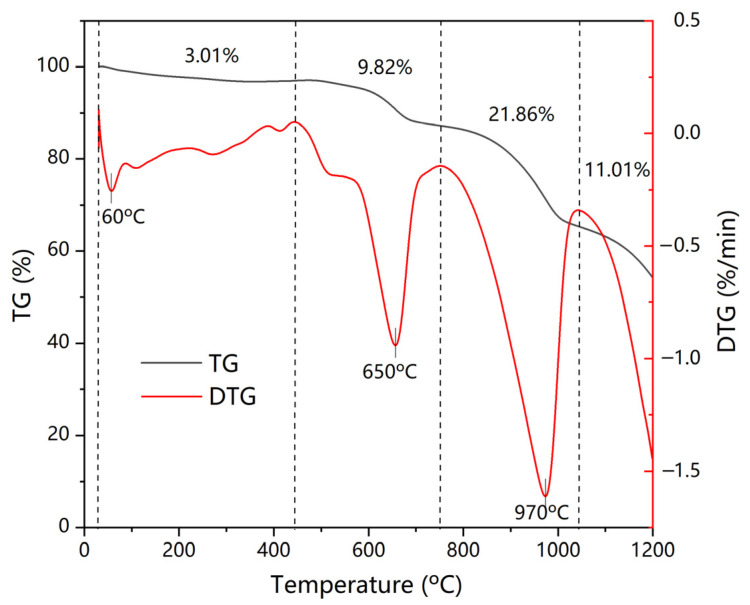
TG-DTG curves of fly ash in N_2_ (Black curve is TG and red curve is DTG).

TG analysis was first conducted to study the thermal behavior of the fly ash as exhibited in Figure 1. The total mass loss in N_2_ reached 45.7% as the temperature reached 1200 °C. The mass loss process could be divided into four stages with three distinct peaks in the DTG curve. The initial mass loss of 3.01% before 450 °C was typically associated with the dehydration of fly ash or the loss of volatile organics [22]. An amount of 9.82% of loss was observed between 450 °C and 750 °C owing to the further pyrolysis of organics and the decomposition of minerals such as CaClOH and Ca(OH)_2_ [23]. The highest amount of mass loss occurred at the third stage (750–1050 °C), which was mostly attributed to the continuing decomposition of inorganic compounds (CaCO_3_, CaSO_4,_ etc.). Wons et al. [24] monitored the release of gas during thermal treatment and found the presence of H_2_O, CO_2,_ and SO_2_ in exhaust at temperatures beyond 800 °C. This showed that chemical compounds, such as CaSO_4_, that were stable at lower temperatures started to decompose. Another 11.01% mass was lost at temperatures between 1050 °C and 1200 °C due to the volatilization of salts, i.e., NaCl, KCl, and CaCl_2_. The results showed that chemical compounds in fly ash underwent complex changes under high temperatures.

### 3.2. Heavy Metal Leaching After Thermal Treatment

According to the thermogravimetric analysis results, significant mass losses were observed at temperatures above 500 °C. Therefore, the effects of thermal treatment on heavy metal leaching toxicity were conducted under temperatures between 500 °C and 1100 °C. Table 1 summarizes the leaching toxicities of heavy metals Cd, Pb, Cu, Zn, Cr, and Ni in fly ash before and after thermal treatment. These six heavy metals had the highest contents (greater than 0.03%) in the raw fly ash, while other metals, such as Sr, Sn, etc., were of trace amount and not of concern.

As shown in Table 1, the leaching toxicities of Cd and Pb were 7.95 mg/L and 12.15 mg/L from the original fly ash. These were much higher than what is required by the National Standard of China (GB 16889-2024) [25], which is the standard for pollution control for landfill sites. The required leaching concentrations of Cd and Pb are set to be less than 0.15 mg/L and 0.25 mg/L, respectively. Although the leaching concentrations of other metals were below what the required amount, overall, the fly ash was still toxic enough to need pre-treatment before landfill.

The results of leaching after thermal treatment demonstrated that thermal treatment was effective in reducing the release of heavy metal. Heavy metal leaching decreased remarkably with the increase in temperature. The leaching concentrations of Cd and Pb were 0.12 mg/L and 0.08 mg/L, respectively, after being thermally treated at 800 °C. The leaching of other heavy metals, such as Cu, Zn, Cr, and Ni, also decreased. In light of the toxicity leaching results, a temperature of greater than 800 °C was needed to treat fly ash and reduce heavy metal toxicity.

### 3.3. Heavy Metal Volatilization

**Figure 2:**
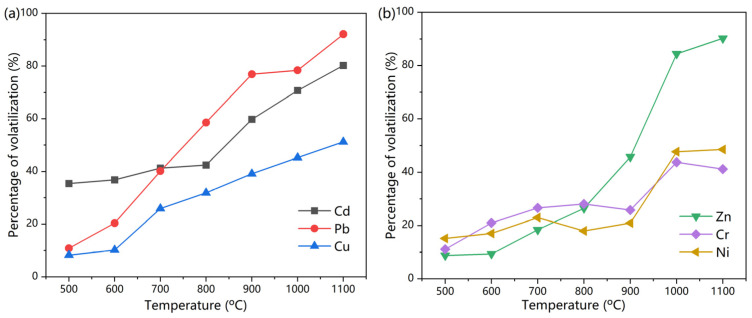
Percentage of volatilization of heavy metals at different temperatures: (**a**) Cd, Pb, Cu (**b**) Zn, Cr, Ni.

As shown in Table 1, leaching of heavy metals decreased remarkably after thermal treatment. However, the reasons for the decreases in leaching toxicity can be two folds. First, it could be caused by the volatilization of heavy metal into the exhaust gas. Thus, the decline in leaching was ascribed to the fact that there are less metals in the fly ash. In their study of the heavy metal behavior during thermal treatment, Han et al. [10] found that the presence of chlorides in fly ash led to significant volatilization of Zn and Pb at a temperature of 1000 °C. Secondly, heavy metals reacted with other compounds in fly ash under high temperatures and were incorporated into the fly ash structure and stabilized [26]. To understand the function of thermal treatment, the percentages of volatilization of heavy metals (Cd, Pb, Cu, Zn, Cr, and Ni) were calculated by Equation (2) based on the contents of heavy metals before and after thermal treatment (Table S2 of Supplementary Materials).

(2) $$\mathrm{PV}\left(\%\right)=\frac{m_{0}c_{0}-\mathrm{mc}}{m_{0}c_{0}}\times 100$$

where PV is the percentage of volatilization; m_0_ is the original fly ash mass; m is the mass after treatment; c_0_ is the original content of heavy metal; and c is the content of heavy metal after treatment. The volatilization of heavy metals is shown in Figure 2.

Thermal treatment led to significant volatilization in heavy metals. Among the six metals studied, 35% of Cd was already volatilized at 500 °C. Other metals were relatively stable at this temperature. As temperature increased, the loss by volatilization reached 58% and 42% at 800 °C for Pb and Cd. By comparison, the volatilization of Cu, Cr, and Ni were much lower at 18–31%. At 1000 °C, about 70% and 78% of Cd and Pb were lost, while the percentages of volatilization were 43–55% for Cu, Cr, and Ni. The behavior of Zn differed slightly. It was stable until 800 °C with less than 26% of volatilization but increased sharply to 54% at 900 °C and to 90% at 1100 °C.

The variation in the degree of volatilization can be explained by the form of heavy metal-containing compounds in the fly ash. For the fly ash in this research, the chloride content via XRF analysis was 14.48%. According to He et al. [27], the chlorides and oxides of Pb and Cd are predominant in fly ash, while Cu, Cr, and Ni were mainly predominant in oxides. The melting points of PbCl_2_ and CdCl_2_ are 501 °C and 568 °C, respectively. These are much lower than those of oxides such as Cr_2_O_3_ (2266 °C) and NiO (1990 °C). Thus, the chlorides of Pb and Cd are much easier to volatilize. Very small amounts of Zn present as ZnCl_2_ in fly ash [28]. The results of Zn volatilization indicate that Zn-containing compounds were stable at temperatures less than 900 °C.

Overall, thermal treatment resulted in the significant volatilization of heavy metals such as Pb and Cd. This could be the main reason for the reduction in leaching toxicities. The high percentage of volatilization also indicated that heavy metal releases during thermal treatment should be of concern. Besides the treatment of flue gas to ensure environmental safety, one of the solutions is to add a stabilization reagent such as SiO_2_ to suppress the release of heavy metal during thermal treatment [29]. In addition, the state of the remaining heavy metals in the fly ash is also important. They have to be in a stable form to prevent leaching.

### 3.4. Heavy Metal Stabilization

**Figure 3:**
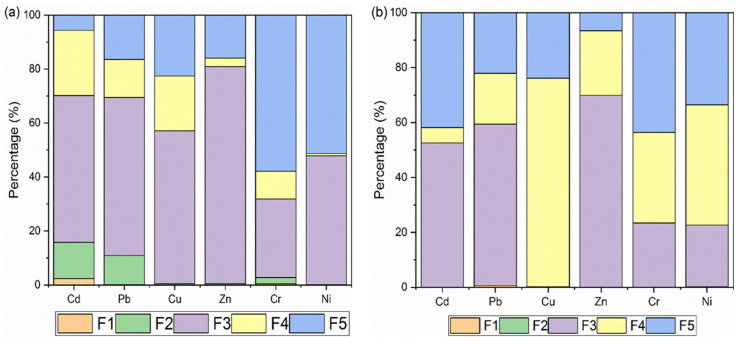
Speciation of heavy metals: (**a**) original fly ash; (**b**) FA800.

The state of heavy metal in fly ash was investigated via speciation using the Tessier method. Figure 3 shows the speciation of heavy metal. The Tessier method divides metals into five speciations (F1–F5) depending on the sequence of extraction [30]. F1 is the exchangeable fraction, while F2–F5 are the carbonate-bound, Fe/Mn oxide-bound, organic-bound, and residual fractions, respectively. The strength of binding increased from F1 to F5. F1 and F2 fractions are considered to be the most loosely bound and readily available. The sum of F1 and F2 are often used as an indicator for the bioavailability of heavy metals. The higher the sum, the higher the risk to the environment.

According to the speciation of the original fly ash (Figure 3a), the fractions of F1 + F2 are the highest for Cd and Pb. This may explain why Cd and Pb had the highest leaching toxicities, as shown in Table 1. These two metals are also the most easily volatilized.

In contrast, more than 98% of the Cu, Zn, Cr, and Ni are in the more strongly bound F3–F5 categories. Specifically, more than 80% of Zn was identified as being Fe/Mn oxide-bound, while amounts greater than 50% of Cr and Ni were found to be in the residual forms. This is also in accordance with results from volatilization. Cr and Ni witnessed the lowest volatilization. This corresponded well with the findings of Wu et al. [31]. They found that the evaporation of heavy metal is closely connected to chemical speciation. The heavy metals in F1 speciation manifested the largest evaporation ratio. In general, evaporation followed the order of F1 > F2 > F3 > F4 > F5.

Speciation results were also obtained for heavy metals after thermal treatment. Figure 3b is the speciation of Cd, Pb, Cu, Zn, Cr, and Ni in fly ash treated at 800 °C. The remaining heavy metals were predominantly in the categories of F3–F5. Taking Cd and Pb as examples, fractions in F1 (exchangeable) and F2 (carbonate-bound) disappeared completely. There were significant increases in the residual fractions. The same also occurred with Cu, Zn, Cr, and Ni. The larger part of these metals was already in the more stable fractions (F3–F5). After thermal treatment, the fractions of F4 + F5 increased while F3 decreased. This shows that thermal treatment was able to stabilize heavy metals even further.

In summary, based on the characterization of volatilization and stabilization behavior, thermal treatment resulted in varying degrees of volatilization of metals. Therefore, the content of heavy metals in fly ash declined. At the same time, the remaining metals were stabilized, which led to the eventual decrease in leaching toxicity.

### 3.5. Phase Transitions via XRD Analysis

**Figure 4:**
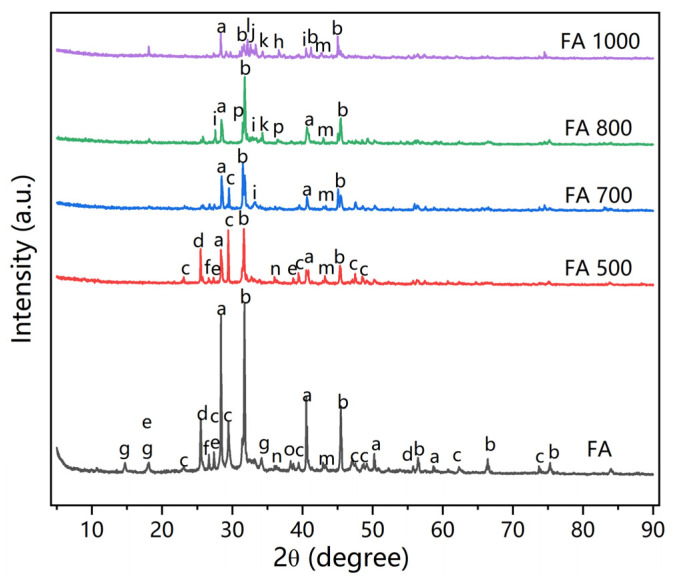
XRD patterns of fly ash before and after thermal treatment (a-KCl, b-NaCl, c-CaCO_3_, d-CaSO_4_, e-CaClOH, f-SiO_2_, g-Ca(OH)_2_, h-FeO, i-Ca_2_SiO_4_, j-Ca_3_Mg(SiO_4_)_2_, k-Ca_3_Fe_2_(SiO_4_)_3_, l-Ca_2_Al_2_SiO_7_, m-MgO, n-Fe_2_O_3_, o-Cu_2_S, p-CuSiO_3_).

The XRD spectra of the original and thermally treated fly ash were next obtained to study the phase transitions with the change in temperature. Figure 4 is the XRD pattern. A number of Ca compounds were found, including Ca(OH)_2_, CaSO_4_, CaClOH, and CaCO_3_. Sharp peaks of NaCl and KCl were also detected. Oxides such as SiO_2_, along with Fe_2_O_3_ and MgO, were also present, which was in line with other studies [32].

Significant changes in the crystal phases were observed after thermal treatment. As temperature increased, the number of crystal compounds declined. However, the chemical structures of the compounds became more complicated. As shown in Figure 4, compounds such as CaClOH, Ca(OH)_2_, CaCO_3_, and CaSO_4_ gradually disappeared as the temperature increased from 500 °C to 1000 °C. The appearance of more complex compounds revealed that decomposition products from the original compounds reacted to form more complicated chemicals, including Ca_3_Mg(SiO_4_)_2_, Ca_3_Fe_2_(SiO_4_)_3_, and Ca_2_Al_2_SiO_7_ [33]. The Cu-containing compound Cu_2_S was detected in the original fly ash, while CuSiO_3_ was present in FA800 and FA1000. This means that heavy metals also participated in the reactions under high temperatures and formed new chemicals. The transitions of crystalline structures showcased that the stabilization of heavy metals was achieved by the breaking up of old compounds and the formation of new compounds. In addition, not all heavy metal compounds were found via XRD analysis. This did not necessarily mean they were not present in the fly ash; it is likely that some metal-containing compounds were amorphous and not detected by XRD.

### 3.6. Changes in Other Elements

The investigation of heavy metal leaching, volatilization, and speciation revealed that heavy metals were volatilized and stabilized by thermal treatment. To further clarify the changes in fly ash, XRF analyses were carried out on the original and thermally treated fly ashes and are listed in Table S3 in the Supplementary Materials. According to the XRF results, the predominant elements in fly ash before and after thermal treatment were Ca, Cl, Na, and K. Compared with the original fly ash, contents of Ca, K, Na, Cl, Fe, Ba, and heavy metals (Cd, Pb, Cu, Zn, Cr, and Ni) dropped while contents of Si, Mn, Mg, Al, and S increased slightly.

**Figure 5:**
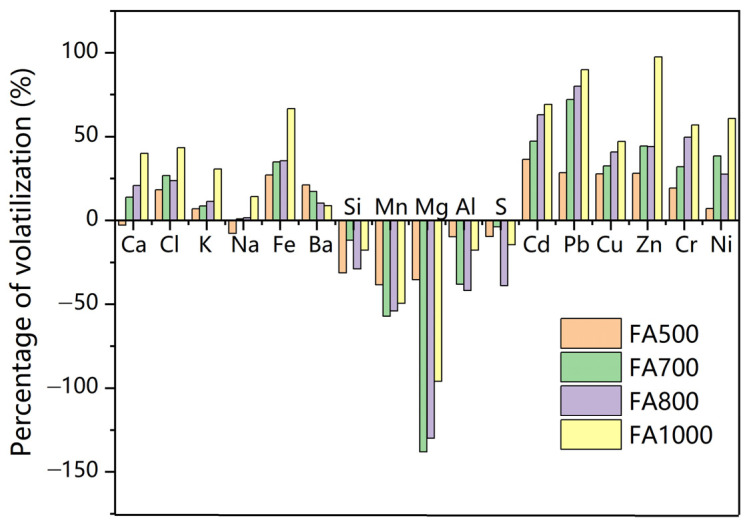
Percentage of volatilization of major elements at different temperatures.

The percentage of volatilization of major elements (content ≥ 0.03%) were also calculated based on Equation (2). Figure 5 is an illustration of the percentage of volatilization of major elements at temperatures of 500 °C, 700 °C, 800 °C, and 1000 °C. Equation (2) is based on mass balance. A positive percentage of volatilization indicates a loss of mass to the gaseous phase. A negative volatilization value, however, indicates that the mass loss of this element was lower than the loss of total mass, that is, the element is enriched in the fly ash.

The volatilization of heavy metals calculated via XRF analysis was, in general, comparable with results via the digestion method (Figure 2). Cd and Pb were more readily volatilized than Cu, Zn, Cr, and Ni.

The elements Ca, Cl, K, Na, Fe, and Ba were also volatilized. The content of Ca in the original fly ash was 43.4%. It decreased to 39.9% at 800 °C (Table S3). Based on the volatilization calculation, 20% of Ca was volatilized. Zhao et al. [34] believed that the loss of Ca could be explained by the decomposition of CaClOH into CaCl_2_ and the evaporation of CaCl_2_ under high temperature. The volatilization of Na, K, and Ba were small before 800 °C, probably because of NaCl, KCl, and BaCl_2_ all have very high boiling points. At temperatures higher than 800 °C, part of the NaCl and KCl could be transformed to form HCl and thus be volatilized [35]. The volatilization of K reached about 11% at 1000 °C. On the other hand, the volatilization of Fe reached 35% at 800 °C and 66% at 1000 °C, since iron chlorides such as FeCl_3_ have a relatively low boiling point (316 °C).

In addition, about 25% of the Cl was lost at 800 °C. The percentage of volatilization reached 42% at 1000 °C. This shows that thermal treatment led to de-chlorination. There are studies showing that chlorides react with oxides to generate chlorine gas at high temperatures [36]. SiO_2_ and Al_2_O_3_ were both found to promote the release of Cl [37]. The chlorine generated reacted with heavy metal oxides, leading to heavy metal volatilization. However, the content of chlorides was still high at about 12% after thermal treatment at 800 °C. Therefore, thermal treatment had a certain effect on dichlorination, but its dichlorination capacity was limited. To increase dichlorination, extra reagents may be needed. Zhao et al. [38] employed iron sulfate as a chlorine depletion reagent to remove chlorides at low temperatures, thus preventing heavy metal volatilization at higher temperatures.

By comparison, the percentages of volatilization were negative for Si, Mn, Mg, Al, and S, indicating that these elements were enriched. This is in accordance with the results obtained via XRD. Oxides, such as SiO_2_ and MgO, which were originally in the fly ash reacted with decomposition products to generate more complicated chemicals and were retained in the fly ash [36]. However, the enrichment effect peaked at around 800 °C. At extremely high temperatures, inorganic compounds, including oxides, started to melt or boil. More compounds could be lost to the gaseous phase.

## 4. Conclusions

Thermal treatment greatly reduced the leaching toxicities of heavy metals. The leaching toxicity met the standard for direct landfill after being treated at 800 °C. The decrease could be attributed to both the volatilization and stabilization of heavy metals. At 800 °C, volatilization amounted to 42%, 58%, 31%, 26%, 28%, and 18% for Cd, Pb, Cu, Zn, Cr, and Ni, respectively. On the other hand, thermal treatment also led to significant stabilization of the remaining metals in the fly ash. The original fly ash was composed of compounds such as CaClOH, Ca(OH)_2_, CaCO_3_, MgO, SiO_2_, Cu_2_S, etc. These compounds decomposed and reacted with each other to form more complex structures, which also resulted in the incorporation of heavy metals. In the end, the speciation of heavy metals after thermal treatment showed that volatilization and stabilization led to the complete disappearance of easily leachable heavy metals in the exchangeable and carbonate-bound fractions. The remaining heavy metals were almost all characterized as Fe/Mn oxide-bound, organic-bound, or as being in residual form. An XRF analysis of fly ash before and after thermal treatment also proved that heavy metals were lost during thermal treatment together with a significant loss of Cl. The phenomenon of dichlorination is in agreement with other studies which have shown that the evaporation of chlorides of heavy metals was one of the causes of loss of heavy metal to the gaseous phase. Besides the decline in leaching toxicity, thermal treatment was also able to enrich elements such as Si, Mn, Mg, and Al in the fly ash, thus enhancing the potential for the reuse of fly ash.

## Figures and Tables

**Table 1:** Heavy metal leaching toxicities.

Heavy Metal (mg/L)		Cd	Pb	Cu	Zn	Cr	Ni
Leaching toxicity	FA	7.95	12.15	32.39	15.78	1.17	0.33
	FA500	3.50	7.60	26.74	10.91	1.26	0.19
	FA600	1.30	5.30	21.92	8.83	1.18	0.16
	FA700	0.41	1.41	18.44	3.12	1.12	0.09
	FA800	0.12	0.08	15.09	1.09	1.04	ND
	FA900	0.08	0.06	3.88	0.10	1.01	ND
	FA1000	0.06	0.05	1.20	0.10	0.78	ND
	FA1100	0.05	0.04	0.95	0.10	0.72	ND
Standard requirement	-	0.15	0.25	40	100	4.5	0.5

ND: not detected.

## Data Availability

The original contributions presented in this study are included in the article/Supplementary Material. Further inquiries can be directed to the corresponding author.

## Supplementary Materials

The following supporting information can be downloaded at https://www.mdpi.com/article/10.3390/ma18061319/s1, Table S1: The protocol of Tessier sequential extraction; Table S2: The content of heavy metals via digestion method; Table S3: The content of elements via XRF (unit: wt%).
