# Supplementary material for: Volatilization and Retention of Metallic and Non-Metallic Elements During Thermal Treatment of Fly Ash
Source: Materials (Basel). 2025 Mar 17;18(6):1319. doi: 10.3390/ma18061319 (PMC11944209; doi:10.3390/ma18061319)
Supplement: Supplementary file 1 [file materials-18-01319-s001.zip › materials-3456169-supplementary.pdf]

Supplementary Materials

Volatilization and Retention of Metallic and Non-Metallic Elements During Thermal Treatment of Fly Ash

Yegui Wang, Weifang Chen, Yifan Chen, Shuyue Zhang and Baoqing Deng *

School of Environment and Architecture, University of Shanghai for Science and Technology, 516 Jun Gong Road, Shanghai 200093, China; 221580132@st.usst.edu.cn (Y.W.); chenweifang@usst.edu.cn (W.C.); 233401991@st.usst.edu.cn (Y.C.); 222291996@st.usst.edu.cn (S.Z.)

* Correspondence: bqdeng@usst.edu.cn

Table S1. The protocol of Tessier Sequential Extraction

Fraction	Parameters of fractionation	Extraction time (h)
Exchangeable	1.0 mol/L $MgCl_2$, pH 7.0, T=25°C	1
Carbonate-bound	1.0 mol/L CH_3COONa, pH 5.0, T=25°C	8
Fe/Mn oxides-bound	0.04 mol/L $NH_2OH·HCl$ in 25% CH_3COOH (v/v), T=96°C	4
Organic-bound	0.02 mol/L HNO_3+30% H_2O_2+3.2 mol/L CH_3COONH_2 in 20% HNO_3 (v/v), pH 2.0, T=85°C	4.5
Residual	68% HNO_3+37% HCl, T=100°C	2

Table S2. The content of heavy metals via digestion method

Fly ash	Heavy metal (wt%)					
	Cd	Pb	Cu	Zn	Cr	Ni
FA	0.030	0.745	0.390	3.210	0.258	0.038
FA500	0.020	0.593	0.369	3.022	0.200	0.033
FA600	0.013	0.530	0.171	2.457	0.092	0.031
FA700	0.013	0.407	0.152	2.419	0.083	0.031
FA800	0.013	0.304	0.126	2.155	0.083	0.033
FA900	0.010	0.172	0.113	1.559	0.092	0.037
FA1000	0.007	0.172	0.116	0.422	0.090	0.029
FA1100	0.004	0.075	0.097	0.097	0.093	0.025

Table S3. The content of elements via XRF (unit: wt%)

Element	FA	FA500	FA700	FA800	FA1000
Ca	43.442	46.084	42.520	39.912	38.656
Cl	14.480	12.210	12.050	12.810	12.168
K	8.200	7.881	8.523	8.430	8.420
Na	6.390	7.100	7.200	7.280	8.120
Fe	3.040	2.290	2.250	2.270	1.505
Ba	0.119	0.097	0.112	0.124	0.161
Si	2.243	3.040	2.850	3.350	3.912
Mn	0.140	0.200	0.250	0.250	0.310
Mg	0.730	1.020	1.975	1.945	2.120
Al	0.900	1.020	1.412	1.480	1.570
S	3.130	3.540	3.695	5.040	5.312
Cd	0.035	0.023	0.021	0.015	0.016
Pb	0.860	0.635	0.274	0.200	0.130
Cu	0.510	0.380	0.391	0.350	0.400
Zn	3.620	2.688	2.289	2.345	0.132
Cr	0.360	0.300	0.278	0.210	0.230
Ni	0.050	0.048	0.035	0.042	0.029
